# Risk factors for complications in patients with ulcerative colitis

**DOI:** 10.1177/2050640615627533

**Published:** 2016-01-19

**Authors:** Christine N Manser, Jan Borovicka, Frank Seibold, Stephan R Vavricka, Peter L Lakatos, Michael Fried, Gerhard Rogler, the investigators of the Swiss Inflammatory Bowel Disease Cohort Study

**Affiliations:** 1 Division of Gastroenterology and Hepatology, University Hospital Zurich, Zurich, Switzerland; 2 Division of Gastroenterology, See-Spital Horgen, Horgen, Switzerland; 3 Department of Gastroenterology and Hepatology, Kantonsspital St Gallen, Switzerland; 4 Crohn-Colitis Centre Bern-Fribourg, Switzerland; 5 Department of Gastroenterology and Hepatology, Triemlispital, Zurich, Switzerland; 6 1st Department of Medicine, Semmelweis University, Budapest, Hungary

**Keywords:** Ulcerative colitis, mesalamine, extraintestinal manifestation, complication, risk factor

## Abstract

**Background:**

Patients with ulcerative colitis may develop extraintestinal manifestations like erythema nodosum or primary sclerosing cholangitis or extraintestinal complications like anaemia, malabsorption or they may have to undergo surgery.

**Objective:**

The aim of this study was to investigate potential risk factors for complications like anaemia, malabsorption or surgery in ulcerative colitis.

**Methods:**

Data on 179 patients with ulcerative colitis were retrieved from our cross-sectional and prospective Swiss Inflammatory Bowel Disease Cohort Study data base for a median observational time of 4.2 years. Data were compared between patients with (*n* = 140) or without (*n* = 39) complications. Gender, age at diagnosis, smoking status, disease extent, delay of diagnosis or therapy, mesalamine (5-ASA) systemic and topical therapy, as well as other medication were analysed as potential impact factors.

**Results:**

In the multivariate regression analysis a delay of 5-ASA treatment by at least two months (odds ratio (OR) 6.21 (95% confidence interval (CI) 2.13–18.14), *p* = 0.001) as well as a delay with other medication with thiopurines (OR 6.48 (95% CI 2.01–20.91), *p* = 0.002) were associated with a higher risk for complications. This significant impact of a delay of 5-ASA therapy was demonstrated for extraintestinal manifestations (EIMs) as well as extraintestinal complications (EICs). Extensive disease as well as therapy with methotrexate showed a significantly increased risk for surgery (extensive disease: OR 2.62 (1.02–6.73), *p* = 0.05, methotrexate: OR 5.36 (1.64–17.58), *p* = 0.006).

**Conclusions:**

A delay of 5-ASA therapy of more than two months in the early stage of ulcerative colitis (UC) constitutes a risk for complications during disease course. Extensive disease is associated with a higher risk for surgery.

## Introduction

Ulcerative colitis (UC) is characterised by relapsing inflammation of the colonic mucosa. With regard to the consensus-guidelines on the diagnosis and management of UC of the European Crohn’s and Colitis Organisation (ECCO) mesalamine (5-ASA) plays an important and central role in the therapeutic management of UC, particularly for the initial therapy.^1^ It is also recommended for maintenance treatment; not only because it has some effect regarding the reduction of relapses but also it may reduce the risk of colorectal cancer in UC patients.^1^ However, recent publications on this issue did not confirm an independent protective effect against colitis associated cancer. Therefore, it may be concluded that the preventive effect of 5-ASA on the risk to develop colorectal cancer might arise from the anti-inflammatory properties and disease control rather than from a specific antitumor effect.^2–5^

Besides medical therapy, proctocolectomy is an effective treatment option for UC. There are basically two main indications for proctocolectomy: the first is elective colectomy due to chronic medically uncontrolled symptoms or high grade dysplasia with a high cancer risk; the second is emergency colectomy due to very severe colitis/toxic megacolon or even perforations in severely ill patients. However, rates of colectomy in UC patients have decreased over the past 50 years.^6^

A recent prospective 10-year follow-up-study showed a generally good prognosis for patients suffering from UC during the first 10 years of disease regarding the colectomy rate as compared to previous reports in literature.^7^ The cumulative colectomy rate of 9.8% in all patients and 19% in patients with initially extensive colitis was lower than previously reported, with colectomy rates published as high as 24% and 28%, or up to 42% in initially extensive UC.^8,9^ Risk factors associated with a higher risk for colectomy were a significant erythrocyte sedimentation rate (ESR) elevation (hazard ratio (HR) 2.94; 95% CI 1.58–5.46) as well as extensive disease at diagnosis (HR 2.98; 95% CI 1.25–7.08). These same risk factors have been already described in earlier reports.^10,11^

UC, however, is not restricted to the colon as many patients develop extraintestinal manifestations (EIMs) such as erythema nodosum, uveitis and primary sclerosing cholangitis (PSC) or extraintestinal complications (EICs) of the disease like anaemia or malabsorption.^12^ Frequencies of developing at least one EIM vary between 6–47%.^13–18^ In the Swiss inflammatory bowel disease (IBD) cohort study the frequency of EIM among UC patients is 30.5% as reported by a recent study by Vavricka et al.^17^ While the investigators reported on disease activity as well as positive IBD family history to be risk factors for ongoing EIM in Crohn’s disease (CD) patients, there were no risk factors identified in UC patients. The study, however, only investigated impact on EIM but not on EIC. Therefore we set up the current study to investigate potential risk factors for complications in UC.

## Material and methods

### Study design

Data were retrieved from the nationwide Swiss IBD Cohort Study (SIBDCS), in which IBD patients from all regions of Switzerland have prospectively been included since 2006.^19^ The cohort study is supported by the Swiss National Science Foundation and approved by the local ethical committees. Patients can be included if diagnosis of CD, UC or indeterminate colitis has been established at least four months prior to inclusion or if disease has recurred during a shorter period of time. Patients have to give written informed consent at the time of inclusion. In addition they need to be permanently resident in Switzerland and/or need coverage by Swiss health insurance. After consent of the patient, a questionnaire on disease history, disease behaviour and medication in past and present is completed. In addition, patients undergo a clinical and laboratory assessment. A yearly follow-up is conducted and patients regularly get questionnaires with clinical and socioeconomic, as well as psychosocial, questions.

UC patients developing one of the following complications were included: anaemia, malabsorption, EIM and colectomy. Types of EIM included were peripheral arthritis, ankylosing spondylitis, uveitis, pyoderma gangrenosum, erythema nodosum, primary sclerosing cholangitis and aphthous stomatitis. Anaemia was defined as a value below the lower limit of normal based on the particular centres’ normal ranges. Malabsorption was defined as a laboratory deficit of vitamin D, zinc and/or iron. Again, a deficit was defined as a value below the lower limit of normal based on the particular centres’ normal ranges. We obtained data about age at diagnosis, smoking status, gender, family history of IBD, history of appendectomy, disease extent, delay of diagnosis or therapy, 5-ASA systemic and topical therapy, as well as other medication (thiopurines, cyclosporine, methotrexate, biologicals).

### Statistical analysis

Clinical data were retrieved from the data centre of the Swiss IBD Cohort Study at the University of Lausanne. These data as well as additional data obtained from a review of the patients’ files were entered into a database (Access 2000, Microsoft Switzerland Ltd Liab. Co, 8304 Wallisellen, Switzerland). The Statistical Package for the Social Sciences (SPSS, Chicago, Illinois, USA; version 21) was used for the statistical analysis.

Crude differences with respect to complications during the course of disease in relation to age at diagnosis, smoking status, gender, family history of IBD, appendectomy, disease extent, delay of diagnosis or therapy, 5-ASA systemic and topical therapy, as well as other medication were assessed using the Pearson χ^2^ test, the Fisher’s exact test (Fisher’s exact test used if strata comprised a sample size ≤5) or the Mann-Whitney *U*-test. A multivariate logistic regression model was calculated including factors with a significance cut-off of *p* < 0.2 in the univariate analysis to identify risk factors for complications.

## Results

At the beginning of 2012, 2490 patients with IBD have been enrolled in the SIBDCS, of whom 909 patients suffered from UC (36.5% of the total). Charts of 328 of these patients at five major centres were reviewed and 179 were eligible for analysis as all relevant data were complete as regards the onset of disease. As the SIBDCS is not an incidence cohort, unfortunately, for a large number of patients the exact data of initial diagnosis and the exact data of start of first medication were not accessible. To avoid a significant recall bias we did not approach those patients for whom we did not have filed data. The final analysis cohort was composed of 66 women (36.9%) and 113 men (63.1%). Of the 179 patients, 140 developed at least one complication during their disease course. Therefore, a group of patients with complications (*n* = 140) and a group of patients without complications (*n* = 39) were defined. Data were available for a median follow-up time of 4.2 years (0–36.7 years; follow-up complication group: 3.9 years (0–36.7 years), follow-up no complication group: 5.0 years (0.2–22.5 years)). The kind of complications, either EIC or EIM are shown in Table 1. Demographic data and baseline characteristics, as well as odds ratios (ORs) for different variables of both groups are shown in Table 2. As can be seen in the univariate analysis, medication with thiopurines and a delay of 5-ASA therapy > 2 months emerged as significant risk factors for any complication. Medication with thiopurines and a delay of 5-ASA therapy > 2 months increased the risk for any complication by 126% and 389%, respectively. Only factors that appeared to have a significant impact on the risk for complications in UC or which showed a trend with *p* < 0.2 in the univariate analysis were included in the multivariate analysis. A delay of 5-ASA therapy > 2 months as well as medication with thiopurines remained significant risk factors for any complication (OR 6.21 (95% CI 2.13–18.14), *p* = 0.001 for a delay of 5-ASA therapy > 2 months and OR 6.48 (95% CI 2.01–20.91), *p* = 0.002 for medication with thiopurines), while extensive disease as well as medication with cyclosporine did not show a significant effect in the multivariate analysis.

**Table 1.** Kinds of complications among 140 patients with ulcerative colitis (UC) developing complications during the course of disease

Complication	Number of patients affected
Extraintestinal complications	115/140 (82.1%)
Anaemia	107/140 (76.4%)
Malabsorption	44/139 (31.7%)
Surgery	26/139 (18.7%)
Extraintestinal manifestation	77/140 (55.0%)
Arthritis	57/140 (40.7%)
Uveitis	22/140 (15.7%)
Pyoderma gangrenosum	4/140 (2.9%)
Erythema nodosum	11/140 (7.9%)
Aphthous stomatitis	7/140 (5.0%)
Ankylosing spondylitis	6/140 (4.3%)
Primary sclerosing cholangitis	9/140 (6.4%)

**Table 2.** Demographic data, patients' characteristics, odds ratios (ORs) and 95% confidence intervals (CIs) of possible risk factors in the univariate (UV) and multivariate (MV) analysis.

Characteristics	Any complication (*n* = 140)	No complication (*n* = 39)	OR (95% CI) (UV)	*p* (UV)	OR (95% CI) (MV)	*p* (MV)
Age at diagnosis (years), mean ± SD	31.9 ± 12.6	31.9 ± 13.6		0.80^a^		
Age at diagnosis (years), range	14–79	15–64				
Male (%)	88 (62.9)	25 (64.1)	1.06 (0.50–2.21)	0.89	0.69 (0.25–1.94)	0.48
Disease duration (years), mean ± SD	6.8 (7.7)	5.8 (5.5)		0.98^a^		
Smoker at diagnosis (%)	28/131 (20.0)	7/36 (17.9)	1.13 (0.45–2.84)	0.80		
Smoker at last follow up (%)	17/136 (12.1)	4/39 (10.3)	1.25 (0.40–3.96)	0.70		
Family history of IBD (%)	5/39 (12.8)	22/140 (15.7)	1.27 (0.45–3.60)	0.66		
Appendectomy (%)	0/39 (0%)	9/140 (6.4%)	^b^			
Delay of diagnosis (months), mean ± SD	8.9 ± 14.4	8.5 ± 13.2		0.26^a^		
Delay of therapy (months), mean ± SD	12.7 ± 19.9	15.2 ± 48.5		0.05^a^		
Delay 5-ASA > 2 months after start of symptoms (%)	65/87 (74.7)	9/23 (39.1)	4.89 (1.85–12.91)	0.001	6.21 (2.13–18.14)	0.001
5-ASA systemic (%)	91/93 (97.8)	23/24 (95.8)	1.98 (0.17–22.78)	0.58^c^		
Initial 5-ASA dose ≥ 2.4 g (%)	68/91 (74.7)	19/23 (82.6)	0.62 (0.19–2.02)	0.43^c^		
5-ASA topical (%)	47/87 (54.0)	12/24 (50.0)	1.18 (0.48–2.90)	0.73		
TNF-inhibitor (%)	33/140 (23.6)	8/39 (20.5)	1.20 (0.50–2.85)	0.69		
Thiopurines (%)	82/140 (58.6)	15/39 (38.5)	2.26 (1.09–4.68)	0.03	6.48 (2.01–20.91)	0.002
Combination TNF/AZA/6-MP (%)	11/140 (7.9)	4/39 (10.3)	0.75 (0.22–2.49)	0.63^c^		
Methotrexate (%)	12/140 (8.6)	2/29 (6.9)	1.73 (0.37–8.10)	0.48^c^		
Cyclosporine (%)	15/140 (10.7)	1/39 (2.6)	4.56 (0.58–35.65)	0.12^c^		
Extensive disease (%)	84/140 (60.0)	18/39 (46.2)	1.75 (0.86–3.58)	0.12	1.53 (0.54–4.33)	0.43

5-ASA: mesalamine; SD: standard deviation; TNF: tumor necrosis factor; AZA: azathioprine; 6-MP: 6-mercaptopurine.

a Mann-Whitney-U test; ^b^OR cannot be calculated due to one field with value ‘0’; ^c^Exact Fisher test.

Subgroup analysis evaluating the impact of possible risk factors on either EIC or EIM revealed a delay of 5-ASA therapy > 2 months to increase the risk for EIC as well as for EIM (EIC: OR 2.38 (95% CI 1.04–5.49), *p* = 0.04; EIM: OR 5.52 (95% CI 2.05–14.85) (see Tables 3 and 4). While therapy with thiopurines was also associated with an increased risk for EIC (OR 1.88 (95% CI 1.01–3.50), *p* = 0.05) therapy with methotrexate showed an increased risk for EIM in the univariate analysis (OR 3.66 (95% CI 1.10–12.15), *p* = 0.03). Family history for IBD and appendectomy also showed an increased risk for EIM (family history: OR 2.61 (95% CI 1.12–6.08), *p* = 0.02, appendectomy: OR 5.00 (95% CI 1.01–24.79), *p* = 0.03). Again multivariate analysis was performed including only factors with significant impact or a trend with a *p* < 0.2 in the univariate analysis. For EIC the delay of 5-ASA therapy > 2 months as well as medication with thiopurine remained significant risk factors (delay of 5-AS therapy: OR 2.51 (95% CI 1.03–6.08), *p* = 0.04, thiopurine: OR 3.79 (95% CI 1.60–9.00), *p* = 0.002). With regard to EIM a delay of 5-ASA therapy > 2 months remained the only significant risk factor (OR 5.38 (95% CI 1.93–15.03), *p* = 0.001). For positive family history of IBD as well as for personal history of appendectomy the multivariate analysis showed at least a trend for an increased risk (family history: OR 3.11 (95% CI 0.96–10.06), *p* = 0.06, appendectomy: OR 7.94 (95% CI 0.77–81.57), *p* = 0.08).

**Table 3.** Distribution, odds ratios (ORs) and 95% confidence intervals (CIs) of possible risk factors in the univariate (UV) and multivariate (MV) analysis with regard to extraintestinal complication (EIC)

Characteristics	EIC (*n* = 115)	No EIC (*n* = 63)	OR (95% CI) (UV)	*p* (UV)	OR (95% CI) (MV)	*p* (MV)
Male	73/115	39/63	0.94 (0.50–1.76)	0.84	0.66 (0.26–1.66)	0.38
Smoker at diagnosis	24/109	11/57	1.18 (0.53–2.62)	0.68		
Smoker at last follow up	15/113	6/61	1.40 (0.52–3.82)	0.51		
Family history of IBD	18/115	9/63	1.11 (0.47–2.65)	0.81		
Appendectomy	6/115	3/63	1.10 (0.27–4.56)	0.89^a^		
Delay 5-ASA > 2 months after start of symptoms	53/71	21/38	2.38 (1.04–5.49)	**0.04**	2.51 (1.03–6.08)	**0.04**
5-ASA systemic	74/76	39/40	0.95 (0.08–10.79)	0.97		
Initial 5-ASA dose ≥ 2.4 g	53/74	33/39	0.46 (0.17–1.26)	0.12	2.90 (1.15–7.29)	0.15
5-ASA topical	36/71	23/39	0.72 (0.33–1.58)	0.41		
TNF-inhibitor	29/115	12/63	1.43 (0.67–3.05)	0.35		
Thiopurines	69/115	28/63	1.88 (1.01–3.50)	**0.05**	3.79 (1.60–9.00)	**0.002**
Combination TNF/AZA/6-MP	10/115	5/63	1.11 (0.36–3.39)	0.86		
Methotrexate	8/115	6/63	0.71 (0.24–2.15)	0.54		
Cyclosporine	14/115	2/63	4.23 (0.93–19.24)	0.05^a^		
Extensive disease	69/115	32/63	1.45 (0.78–2.70)	0.24		

5-ASA: mesalamine; TNF: tumor necrosis factor; AZA: azathioprine; 6-MP: 6-mercaptopurine. ^a^Exact Fisher test.

**Table 4.** Distribution, odds ratios (ORs) and 95% confidence intervals (CIs) of possible risk factors in the univariate (UV) and multivariate (MV) analysis with regard to extraintestinal manifestation (EIM)

Characteristics	EIM (*n* = 77)	No EIM (*n* = 102)	OR (95% CI) (UV)	*p* (UV)	OR (95% CI) (MV)	*p* (MV)
Male	32/77	34/102	1.42 (0.77–2.62)	0.26	1.25 (0.50–3.12)	0.63
Smoker at diagnosis	17/77	18/96	1.36 (0.65–2.88)	0.42		
Smoker at last follow-up	8/74	13/101	0.82 (0.32–2.09)	0.70		
Family history of IBD	17/77	10/102	2.61 (1.12–6.08)	**0.02**	3.11 (0.96–10.05)	0.06
Appendectomy	7/77	2/102	5.00 (1.01–24.79)	**0.03**	7.94 (0.77–81.57)	0.08
Delay 5-ASA > 2 months after start of symptoms	40/46	35/64	5.52 (2.05–14.85)	**0.0001**	5.38 (1.93–15.03)	**0.001**
5-ASA systemic	48/49	66/68	1.46 (0.13–16.51)	0.76		
Initial 5-ASA dose ≥ 2.4 g	38/48	49/66	1.32 (0.54–3.21)	0.54		
5-ASA topical	27/47	32/64	1.35 (0.63–2.88)	0.44		
TNF-inhibitor	16/77	25/102	0.81 (0.40–1.65)	0.56		
Thiopurines	41/77	56/102	0.94 (0.52–1.69)	0.83		
Combination TNF/AZA/6-MP	4/77	11/102	0.45 (0.14–1.48)	0.18	0.32 (0.03–4.22)	0.39
Methotrexate	10/77	4/102	3.66 (1.10–12.15)	**0.03^a^**	6.84 (0.59–78.75)	0.12
Cyclosporine	8/77	8/102	1.36 (0.49–3.81)	0.56		
Extensive disease	47/77	55/102	1.34 (0.74–2.44)	0.34		

5-ASA: mesalamine; IBD: inflammatory bowel disease; TNF: tumor necrosis factor; AZA: azathioprine; 6-MP: 6-mercaptopurine. ^a^Exact Fisher test.

The risk for surgery was increased by an extensive disease course (OR 2.50 (95% CI 1.00–6.26), *p* = 0.05) as well as therapy with methotrexate (OR 5.07 (95% CI 1.60–16.07), *p* = 0.003). Besides a trend was observed for an increased risk among patients having had an appendectomy (OR 3.00 (95% CI 0.70–12.81), *p* = 0.12). There also was a trend towards a protective effect for male sex (OR 0.54 (95% CI 0.22–1.36), *p* = 0.19). In the multivariate analysis, however, only extensive disease as well as therapy with methotrexate showed a significantly increased risk (extensive disease: OR 2.62 (95% CI 1.02–6.73), p = 0.05, methotrexate: OR 5.36 (95% CI 1.64–17.58), *p* = 0.006).

## Discussion

This cross-sectional study with a prospective follow-up in a very well characterised patient population, the SIBDCS is the first study reporting on risk factors for complications in UC patients. We demonstrate that a delay in the onset of 5-ASA therapy early after diagnosis of UC is a risk factor for development of complications. This underlines the importance of 5-ASA therapy at disease onset. A diagnostic delay in UC has been previously estimated to be less relevant in UC as compared to CD.^20,21^

Why are complications in UC patients more likely to occur when the start of 5-ASA treatment is delayed >2 months? One possible explanation is that a late therapy start may lead to more extended disease and more severe inflammation. In a South Korean study, 27.6% of all UC patients experienced proximal disease extension. Those patients with extended disease finally had more severe inflammation as compared to patients with distal disease as assessed by Mayo subscores.^22^ This was associated with chronic, continuous disease activation. In a Hungarian IBD cohort, the probability of proximal disease extension was 12.7%.^23^ Both the Korean and Hungarian cohorts however, do not provide data as to whether the disease extension was associated with a delay in therapy.

In line with our data, a recent publication from the UK showed that 5-ASA users in the investigated patient sample were less likely to require a colectomy (OR 0.35, 95% CI 0.28–0.44).^24^ In this cohort the administration of thiopurine for more than 12 months was associated with a 71% reduction in risk of colectomy (OR 0.29, 95% CI 0.21–0.40). However, an early start of thiopurines did not show any additional benefit. Thus it seems that there is a clear difference between early 5-ASA and early thiopurine use with respect to long-term disease outcome.

Higher doses of 5-ASA more quickly evoke healing of inflammation and therefore also have a positive effect on avoiding complications. The benefit of higher doses of 5-ASA on mucosal healing has been demonstrated in the past.^25–27^ Among our patients, however, there was only a trend observed towards a risk reduction for EIC in the case of an initial dose of 5-ASA ≥ 2.4 g (multivariate analysis OR 2.90 (95% CI 1.15–7.29), *p* = 0.15).

Some of the investigated complications, like anaemia, correlate with intestinal inflammation, either due to anaemia of chronic disease or due to iron deficiency. However, subgroup analysis regarding anaemia as a complication on its own, did not reveal a significant impact of delay of therapy, although, a trend was observed (univariate analysis delay of 5-ASA therapy > 2 months OR 2.08 (95% CI 0.92–4.71), *p* = 0.08). Besides a trend was observed towards a higher risk among patients on therapy with thiopurines (univariate analysis OR 1.71 (95% CI 0.94–3.14), *p* = 0.08) as well as cyclosporine (univariate analysis OR 3.14 (95% CI 0.86–11.43), *p* = 0.07). However, and this is a limit of our study, we did not correlate these findings to clinical or endoscopical disease activity.

Many studies on the impact of 5-ASA on the course of disease focus on a possible chemoprevention of 5-ASA with regard to development of colorectal cancer. A very recent meta-analysis on this issue by Zhao et al. including 17 studies with 1508 UC patients with colorectal neoplasia and a total of 20,193 patients reported of a reduced risk for colorectal cancer in UC patients when on therapy with 5-ASA (OR 0.63 (95% CI 0.48–0.84)).^28^ They, however, point out that there is a considerable methodological heterogeneity among the selected studies. Other authors therefore come to the conclusion that, especially, recent data have failed to show a chemopreventive effect of 5-ASA in colorectal cancer.^29^ Among our patients we might need a longer follow-up to answer the question as to whether a therapy with 5-ASA in the early course of disease might prevent development of colorectal cancer.
